# Sicilian Byzantine Icons through the Use of Non-Invasive Imaging Techniques and Optical Spectroscopy: The Case of the *Madonna dell’Elemosina*

**DOI:** 10.3390/molecules26247595

**Published:** 2021-12-15

**Authors:** Francesco Armetta, Gabriella Chirco, Fabrizio Lo Celso, Veronica Ciaramitaro, Eugenio Caponetti, Massimo Midiri, Giuseppe Lo Re, Vladimir Gaishun, Dmitry Kovalenko, Alina Semchenko, Dariusz Hreniak, Maria Luisa Saladino

**Affiliations:** 1 Department of Biological, Chemical and Pharmaceutical Sciences and Technologies—STEBICEF, University of Palermo, Viale delle Scienze Ed. 17, I-90128 Palermo, Italy; ciaramitaroveronica@gmail.com (V.C.); eugenio.caponetti@unipa.it (E.C.); 2 Department Culture e Società, University of Palermo, Viale delle Scienze Ed. 15, I-90128 Palermo, Italy; gabrychirco@gmail.com; 3 Department of Physics and Chemistry “E. Segre”, University of Palermo, Viale delle Scienze Ed. 18, I-90128 Palermo, Italy; fabrizio.locelso@unipa.it; 4 Labor Artis C.R. Diagnostica S.R.L., I-90145 Palermo, Italy; 5 Department of Biomedicine, Neuroscience and Advanced Diagnostics, University of Palermo, Via del Vespro 129, I-90127 Palermo, Italy; massimo.midiri@unipa.it (M.M.); giuseppe.lore01@unipa.it (G.L.R.); 6 Faculty of Physics, Francisk Skorina Gomel State University, Soviet Str. 104, 246019 Gomel, Belarus; vgaishun@gsu.by (V.G.); dkov@gsu.by (D.K.); alina@gsu.by (A.S.); 7 Institute of Low Temperature and Structure Research, Polish Academy of Science, ul. Okólna 2, 50-422 Wrocław, Poland; d.hreniak@intibs.pl

**Keywords:** Byzantine Icons, imaging techniques, pigments, X-ray tomography

## Abstract

The iconographic heritage is one of the treasures of Byzantine art that have enriched the south of Italy, and Sicily in particular, since the early 16th century. In this work, the investigations of a Sicilian Icon of Greek-Byzantine origin, the *Madonna dell’Elemosina*, is reported for the first time. The study was carried out using mainly non-invasive imaging techniques (photography in reflectance and grazing visible light, UV fluorescence, infrared reflectography, radiography, and computed tomography) and spectroscopic techniques (X-ray fluorescence and infrared spectroscopy). The identification of the constituent materials provides a decisive contribution to the correct historical and artistic placement of the Icon, a treasure of the Eastern European historical community in Sicily. Some hidden details have also been highlighted. Most importantly, the information obtained enables us to define its conservation state, the presence of foreign materials, and to direct its protection and restoration.

## 1. Introduction

In the Byzantine tradition, the Icons represent documents of historical, theological, and philosophical as well as artistic interest. For the Eastern faithful, the Icon is *anàmnesis* (remembrance–recall), it is *kèrisma* (announcement–catechesis), it is *theoria* (contemplation–prayer), it is a reference to tradition, it is an announcement–declaration of a presence, and it is contemplation–involvement vital for a journey of hope. Therefore, the study of the Byzantine Icons constitutes an immense historical heritage, and their safeguarding contributes to the knowledge of the religious customs of ancient civilizations. The main current topics are the understanding of the techniques of realization in a given historical period, the originality of the artefact, and the definition of their state of conservation.

To our knowledge, in the last few years, some studies have been conducted about the investigation of Icons [1,2], mainly from Albania (post-Byzantine icons) or Russia, or of Lipovian style [3,4,5]. Most of the investigations were performed by applying a non-invasive approach to identify the pigments [6,7] or to compare some Icons in order to give the proper attribution to a particular iconographer [8,9,10,11,12,13,14,15]. Moreover, this output is very small compared to the large number of unstudied artworks that are in need of conservation, and no similar work has been published about the Icons saved in Belarus, Poland, or Italy. For this reason, in the ICONS Project, a cooperation between three different research groups from the Republic of Belarus, Poland, and Italy, started to investigate some Icons saved in each country (Ref. No. 1206.008-19, financed by the CEI Know-how Exchange Programme). The goal of the research was the understanding of the painting techniques used by the iconographers in a given historical period, the originality of the Icon itself, and the definition of their conservation states. Among the cultural heritage sites and museums in Belarus, the Vetka Museum of Old Believer Faith and Belarusian Traditions in the Gomel region is evidence of the history of the country because it has a unique collection of the Old Believers’ Icons, manuscripts, and early printed books of the 16–19th centuries, and others [16], and it presents the art, culture, and creativity of several folk traditions of the region in the southeast of Belarus. In Poland, the National Museum in Krakow saves the oldest and most precious collections of old Orthodox paintings and Icons in Central Europe [17] (from the 15th to 17th century under the Jagiellonian dynasty’s rule), originating from the territory of the historical Orthodox Diocese of Przemyśl within the borders of the Polish Kingdom [18]. In Italy, in some small towns, especially in the south, many Icons are saved and preserved by each local community which, since the early 16th century, consider the sacred images the prestigious heritage of Greek art or ancient Byzantine tradition, testimony, and treasure of the east European art and spirituality of their historical community [19,20].

Since the material knowledge contributes to the correct placement and “reading” of an ancient artefact, for the first time, the investigation of a Sicilian Icon of Greek-Byzantine origin is here reported. The goal of the investigation was to know its constituents (pigments, binders, and varnishes) as well as the possible degradation products so to deepen the knowledge of the painting techniques used by the iconographers as a support for art historians. The investigation of the Icon *Madonna dell’Elemosina* was carried out considering the principle of non-invasiveness. The diagnostic studies were carried out following this virtuous protocol based on the use of imaging and spectroscopic techniques, which are all non-invasive and mainly applicable in situ by using portable instruments [21,22,23,24,25]. First of all, a multispectral investigation was performed by illuminating the Icon with different lights and recording photographs with a camera equipped with interchangeable filters so to obtain several images in reflectance and grazing visible light, in UV fluorescence, and in infrared reflectography. The above techniques, together with the radiography and computed tomography, gave a complete overview of the Icon in each of its layers. Spectroscopic techniques (X-ray fluorescence and infrared spectroscopy) were thus used to obtain information on the nature of the inorganic pigments and the organic compounds in some areas selected on the basis of the obtained images. The main advantage of this choice lies in the possibility of carrying out a large number of measurements on a work of art with the guarantee of the respect for its integrity [26,27].

## 2. The Icon *Madonna dell’Elemosina*

In 1482, following the victory of the Muslim Turks in Greek-Albanian land, a colony of refugees coming from Scutari and led by Cesare De Masi landed in Sicily, bringing with them its most precious treasure: the Byzantine Icon of the Mother of God, “Eleùsa”. The final destination of the small group of exiles was Palermo (Italy), where they planned to join their other countrymen in the current Piana degli Albanesi (once, Piana dei Greci). During their journey, the exiles stopped about 30 km from Catania, in a field called “Callicari”. Here, according to tradition, after having planted the camp, they hung the sacred Icon on a fig tree. In the morning, when it was time to resume their journey, the exiles found their Icon entirely entangled among the branches of the fig tree, which had grown overnight. The event was interpreted as the will of the Mother of God, who had guided them on their journey, to remain in that place, where the small group could find a new homeland.

On the basis of the tradition, the Icon *Madonna dell’Elemosina*, today saved at Basilica Santuario S. Maria dell’Elemosina (Biancavilla (CT), Italy), was realized by the egg tempera technique on cedar wood at the beginning of the 14th century and belongs to the Greek-Albanian school. It has been the object of uninterrupted veneration since the end of the 15th century and has received the title of “Guardian of the people of Etna”.

The Icon was restored two times, in 1948 and 1998. Although not very visible, two (of the three) stars, typical of Marian iconography and a symbol of divinity, are present. The letters (inscriptions on the Icon), visible today, were added in 1998 after being canceled in the restoration of 1948.

## 3. Materials and Methods

The photos, in diffused visible light, in grazing light, and in UV fluorescence, were acquired by using a Canon EOS 500D camera with an 18–55 mm lens. Two visible lamps placed with an angle of 45° were used to illuminate the Icon to obtain the photos in diffused visible light. The photos in grazing light were taken by illuminating the Icon with a visible light at 20° with respect to the surface in order to increase the contrast between the illuminated areas and those in shadow, and to eliminate the light diffusion effects, accentuating all the defects of the surface. The photos in UV fluorescence were taken in a dark room by illuminating the Icon with an ultraviolet source (wood lamp 27 E, 160 W). The photos in infrared (IR reflectography) were taken in the range 400–1100 nm with a Nikon D100 camera.

The radiograms were collected with an image receptor distance (SID) of 140 cm. The X-ray source was used with a tube voltage of 50 kV, a current of 100 mA, and a time exposure of 0.10 s. The FOV is 45 × 35. A CT multidetector (128 rows) was also acquired with the following parameters: 120 kV, 150 mA, slice thickness 0.6 mm, overlap 0.35 mm, pitch 0.6, kernel B20f. The obtained images were also reconstructed with MPR, MIP, and 3DVR reconstructions using an advanced workstation (Horos 4.0.0. RC5).

The XRF measurements were performed in situ by placing a portable spectrometer Tracer III SD Bruker AXS in contact with the selected colored area of the Icon. The source was a Rhodium Target X-Ray tube operating at 40 kV and 11 mA and an acquisition time of 30 s. The detector was a 10 mm^2^ silicon drift X-Flash with Peltier cooling system and a resolution of 145 eV at 100,000 cps. The S1PXRF^®^ software managed the data acquisition. The spectral assignment of the characteristic peaks of each element was carried out using the database contained in the ARTAX 8 software. In each spectrum, the signals of rhodium (Rh) and argon (Ar), due to the source and the atmosphere, respectively, were present.

The reflectance FT-IR spectra were acquired by using the portable Bruker ALPHA FT-IR spectrometer equipped with an external reflection QuickSnapTM module. Spectra were acquired between 3500 and 360 cm^−1^ with a resolution of 4 cm^−1^, averaging 60 scans for each measurement. All reflectance spectra were processed by using OPUS 7.5 software; each spectrum was converted to pseudo-absorbance (Log R) units in order to compare the spectrum with a database of reflectance reference spectra.

## 4. Results and Discussion

### 4.1. Imaging Techniques

**Figure 1:**
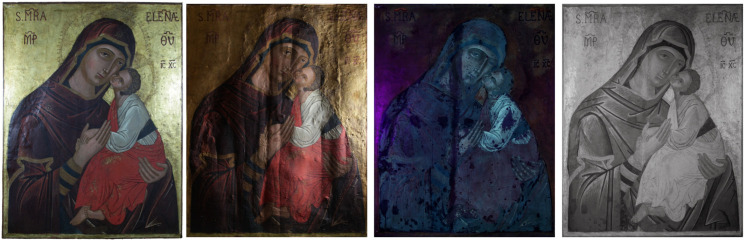
The Icon *Madonna dell’Elemosina* (Biancavilla (CT), Italy). From (**left**) to (**right**): photo in diffused vis light; vis grazing light; UV fluorescence; IR reflectography.

The images obtained in diffused visible light (vis), in grazing visible light, UV fluorescence, and IR reflectography are shown in Figure 1.

**Figure 2:**
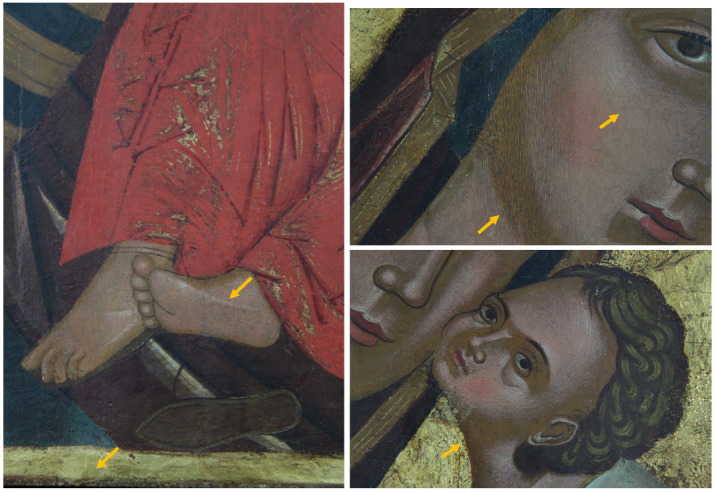
Vis: Detail, feet of the Child Jesus in which the colors overlap, the mission gilding, and a retouching are observed (**left**); face of the Child Jesus (**right up**); face of the Madonna, chromatic integration (**right down**).

The painting, 67 cm × 86 cm × 2 cm in size, is in a fairly good state of conservation. The image is flat and not plastic, which are typical canons of Byzantine art. The pictorial layer is full-bodied and opaque in the larger backgrounds and along the contours of the garments’ folds, while the details, shadows, and the brighter areas are created by overlapping a more fluid color. The decorations, made with gold leaf, were performed with the mission technique (e.g., fringes on the mantle of the Madonna and decorations on the mantle of the child, Figure 2). The gold background is made with the gouache gilding technique with a brown-red bolus. Concerning the conservation aspect, small gaps along the perimeter of the work are present, while the gold background is abraded in the area of the halos and in some areas of the perimeter. In several areas, some repainting/overpainting (indicated as arrows), made with the hatching technique, are present (Figure 2, Figures S1 and S2). Finally, on the right side of the Icon, an imprint of lipstick is evident (Figure S3).

**Figure 3:**
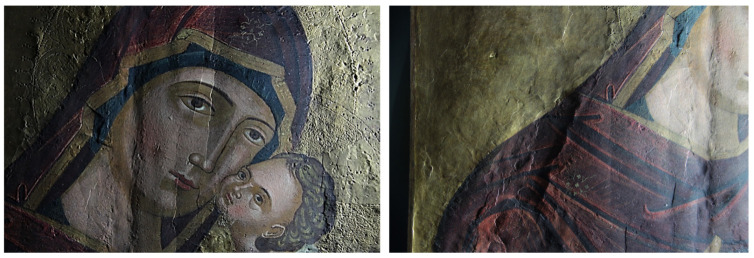
Vis grazing: Detail, face of the Madonna and Child Jesus in which are lesions and an unevenness of the surface, some brushstrokes in relief, and the incisions of the halos can be observed (**left**); irregularities in the pattern of the pictorial surface (**right**).

Details obtained in visible grazing light are shown in Figure 3 and Figure S4. They clearly highlight the depressions, in the vertical direction, of the surface due to the damage of the support and to the joint between the two axes of which the support is made. In fact, a vertical depression affecting the entire length of the wooden support is evident in the center. This area undergoes the greatest stress due to the natural movements of the wood, which affects the painting layer, causing swelling, lifting, and detachment. In the light of this fact, the greatest number of pictorial additions made during the previous restoration work can be found in this area. The planks have a slight bow. The left area at the height of the Madonna’s shoulder is particularly compromised. The engravings of the halos, made with a punch for the smaller holes and with a burin for the larger ones, can be observed in detail. Other incisions are identified in the area of the inscription tracing the outline of some alphabet letters, which are not present today and do not correspond to those visible, but which are probably the prints of the original ones.

**Figure 4:**
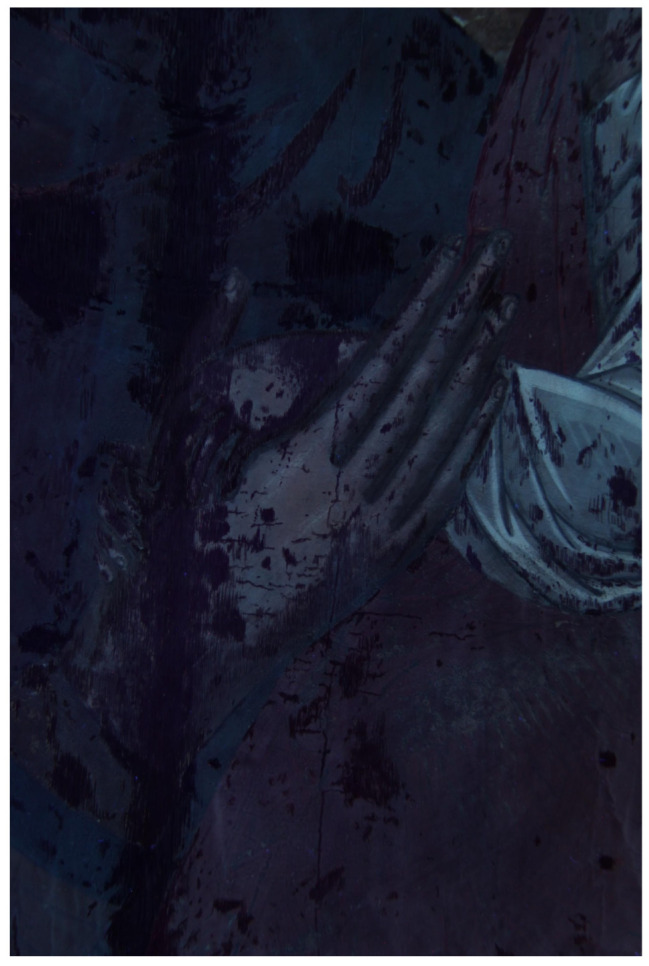
UV fluorescence: Detail, hands of the Madonna and Child Jesus, and numerous pictorial retouches can be observed.

The images obtained in UV fluorescence are shown in Figure 4 and Figure S5. In the area of the Madonna and the Child, the fluorescence is bluish, indicative of a varnish of a synthetic nature and which is not original. On the gold background, the fluorescence is darker and unevenly distributed, which could be due to a substance used to age the non-original gold leaf applied to the gaps in the background. It is not present in the area where the inscriptions are present. Furthermore, numerous retouches are observed affecting the entire pictorial surface, in particular in the vertical direction along the entire length of the panel in the area of the joint between the axes of the support. In the center, along the same line, five areas of repainting with a circular shape are highlighted, which identify the heads of nails. A very strong orange fluorescence, attributable to a previous symbol canceled during the last restoration in 1998, is observed under the symbols MRA and ELENÆ. Similar observations are reported in the literature [28,29].

The infrared reflectography images are shown in Figure 1 and Figures S6–S8. Some details not well visible to the naked eye are highlighted but no difference with the visible image is observed. The outlines of the two figures and of details, in particular of the garments and the veil, are realized with a thick-tipped brush to define the margins. As already observed through the illumination under UV light, the areas of lacunae, repainting, and retouching are highlighted, in particular on the shoulder of the Madonna and around the feet of the infant Jesus where the pictorial layer is abraded.

The X-ray radiography of the Icon is shown in Figure S9. The wooden support was made using two axes of different widths, and they are affected by numerous cracks of different depths and by the signs of a consistent attack of xylophagous organisms. A knot in the wood forms a bulge on the front of the work under the elbow of the Child. In several places, gauzes were applied to the cracks to reinforce the injured areas. Traces of glue, probably used to restore the wood, can be observed along many of the cracks. The support was fixed with a fixed parking lot consisting of five vertical and five horizontal axes, having different and being positioned at different distances. This practice, widely used in the past for the restoration of paintings, a very invasive intervention, is the cause of the cracks observed on the support because it does not allow the natural movement of the wood. The modern wooden lath, surrounding the table, was fixed with nails to the boards of the parquet. The central axis, probably original, was nailed to the work using round-headed nails, some of which are clearly visible.

**Figure 5:**
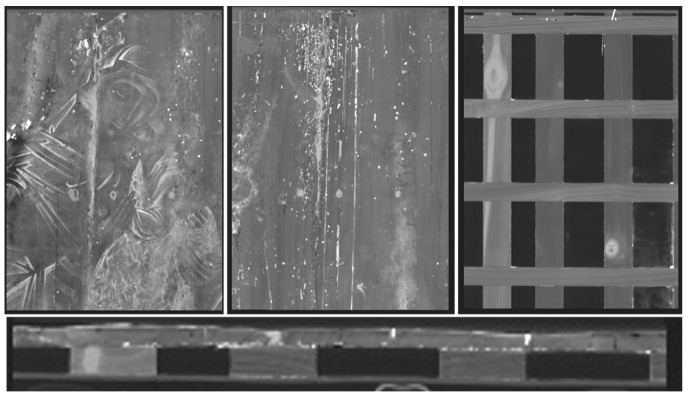
CT scan. (**Top from left to right**) front of the Icon, towards the Icon, the parking area; (**down**) the lower and upper section.

**Figure 6:**
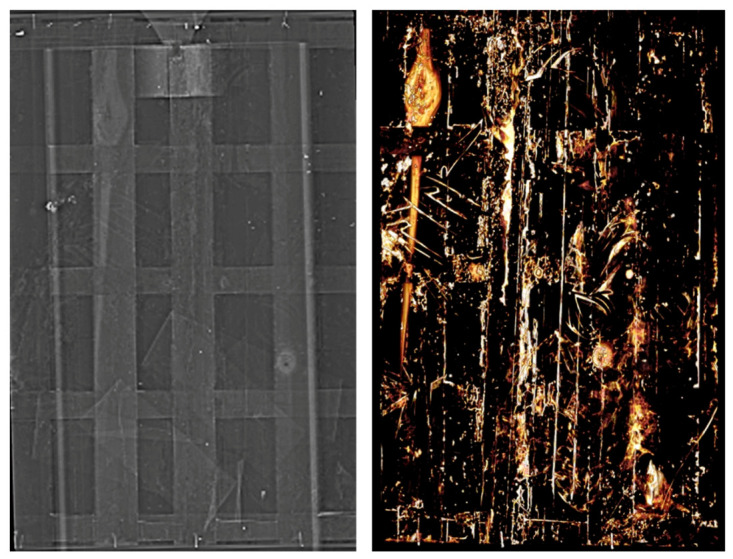
CT scan. (**Left**) wide bandage folded several times in a zig zag and a trapezoidal wooden insert with a hook; (**right**) Greek letters.

The images obtained by CT scan are shown in Figure 5 and Figure 6 and highlight the different layers of the Icon. As evidenced by the radiography, today it is composed of the painted panel, of the crosspieces constituting the intervention of the parking lot, and of a closing panel fixed to them. Two different types of wood, used for the crosspieces and for the support, are evident. In the lower part, between the crosspieces and the table, a 9.5 cm wide bandage can be seen, folded several times in a zig zag pattern, inserted during a previous restoration, while in the upper part a trapezoidal wooden insert with a metal hook is present between the panel and the table. The thickness of the Icon is 16 mm, of which about 2 mm is the preparation layer and the paint film. The thickness of the crosspieces is 25 mm, while the total thickness is 43 mm.

The panel of the painting is composed of two wooden planks of subradial sections of different widths (not measurable). The scrupulous choice of the subradial cut is synonymous with the accuracy applied in the realization of the Icon, being that this kind of cut of the wood more stable and therefore less subject to deformation. Throughout the table, numerous walkways that preferentially follow the grain of the wood are present. On the back of the panel of the painting, the stuccos applied during a previous restoration can be observed.

In the center of the painting, along the same line, on the robes of the Madonna and Child, three holes can be observed which were inserted from the front to fix the central crosspiece, as hypothesized by the UV fluorescence. However, they may also have been used in the past to attach gold decorations during the processions or exhibitions of the Icon to the devotion of the faithful. On the back of the original Icon table, on the right side, some Greek-Byzantine characters are evidenced, probably engraved with a metal point, which are not visible when looking at the Icon today due to a second table supporting the one bearing surface.

The images confirm that the Child’s robe was made with cinnabar, in accordance with XRF spectroscopy (see the next paragraph), as well as some decorations of the Madonna’s robe and mantle.

### 4.2. Spectroscopic Techniques

The maps of the area, analysed by XRF and IR spectroscopy, are shown in Figure S10. Table 1 shows the identified elements and the estimated pigments for each analyzed area.

The ubiquitous presence of lead (Pb) signals is indicative of the presence of white lead (basic lead carbonate (2PbCO_3_)_2_·Pb(OH)_2_), used in the preparation mixture or as a primer layer, which is in strong accordance with the results obtained by the chemical analysis of other Believer Icons from Poland that found white lead as preparation layer for all the investigated Icons [30]. In some dark red areas the lead amount is higher, indicating the possible presence of lead oxide (minium), which can become darker after the degradation.

The presence of calcium (Ca) and strontium (Sr) in all the analyzed area is attributable to the gypsum used for the preparation layer. The presence of strontium in small quantities is associated with celestite, a mineral often found together with gypsum [17,31,32].

The presence of iron (Fe) and manganese (Mn) in points P2 and P4 is ascribable to umber (iron oxide hydrated Fe_2_O_3_∙H_2_O with 8–12% manganese dioxide MnO_2_) or burnt umber.

The presence of mercury (Hg) in red and in the incarnate is indicative of the use of cinnabar (HgS).

The blue areas are characterized by the presence of a copper-based pigment, but the identification of the pigment requires an invasive investigation which does not respect the integrity of the Icon, so is out of the scope of this study. However, on the basis of the literature [33], we could hypothesize that it could be azurite.

The concomitant presence of different pigments in the same area is indicative of a mixing of different pigments or of the various color layers’ overlays.

The gilding was made with pure gold (Au) on a bolus, consisting of a mixture of iron oxide and aluminium silicates such as kaolinite or other clay minerals necessary for the adhesion of the metal leaf to the support [27,34].

The similarities found between the FT-IR spectra (in the stretching zone of the C-H (3000–2800 cm^−1^ and below 1800 cm^−1^, Figure S11)) allow us to state that the nature of the analyzed surface is similar in all the analyzed areas, which is in agreement with what was observed under UV light. Alcoholic groups with a large peak centered at 3400 cm^−1^ (stretching –OH aliphatic), C–H stretching at 2926 and 2859 cm^−1^ and at 1726 cm^−1^ (aldehyde/aliphatic ketone C–O–C, carboxylic acid stretching), and ester, acid, and alcoholic groups at 1250, 1165, and 1060 cm^−1^, respectively, are present [35], ascribable to the shellac resin. Furthermore, the presence of the double peak at 730 and 720 cm^−1^ is ascribable to a partially crystalline hydrocarbon chain, Ref. [36] suggesting a shellac–wax coupling used as a protective layer.

## 5. Conclusions

The results presented in this paper are considered of great importance, in particular for deepening the knowledge on the technique of making the Orthodox-Byzantine Icons. The details, the iconographic details, and the conservation problems were highlighted by a combination of visible light image techniques in visible and grazing light photography. Observation under UV light allowed us to examine the nature and state of the surface and the location of pictorial retouching. Subsequently, examination through infrared reflectography provided information on the painting techniques, and allowed us to highlight the areas of lacunae, repainting, and retouching. No preparatory drawings and no regrets were found.

As far as the painting technique is concerned, the Icon was painted by spreading the pigments directly onto a preparation and primer layer of about 2 mm. The outlines of the two figures and of the details were realized with a thick-tipped brush to define the margins. The spectroscopic techniques (X-ray fluorescence and infrared spectroscopy) were useful for identifying the palette used by the iconographers and the gold coating. The presence of cinnabar, umber, a copper-based pigment, and white lead is in line with those of analogous Icons investigated by other authors. On the other hand, the presence of a mixture of shellac and beeswax on the surface is an indication of a recent protection of the paint layer, necessary due to the color losses. Radiography and CT provided information about the wooden support and its conservation state and revealed the hidden structures and some integrations made in previous restorations.

Radiographs and the CT scan have clearly shown that the investigated Icon currently consists of the painted panel, the crosspieces that make up the parquet, and a closing panel that is attached to them. On the back of the table, on the right side, several hidden Greek letters were also discovered on the reverse right, the meaning of which still requires additional research. Overall, it can be said that the Icon is in fairly good condition.

Based on the investigation, no discriminatory elements were identified for a precise temporary location of its completion. The pigments identified, as well as the technique, are consistent with an earlier dating between the 14th and 18th centuries, given the presence of pigments such as cinnabar.

Moreover, in terms of training and the transfer of know-how, the impact of carrying out the presented research in particular, and the ICONS project in general, has been very relevant, both from a historical, and artistic and scientific point of view. Importantly, it should also be stressed that the research methodology presented in this paper can be successfully applied to the analysis of Icons from other areas of Orthodox Church culture, including the Old Believer faith community. The complementary skills and techniques of the three partners were used to learn about the materials constituting the Icon and the main images and spectroscopic techniques used in the field. However, the main part of the project was the transfer of knowledge and training in the archaeometric in order to gain an understanding of artefacts of artistic and archaeological interest. Due to the COVID-19 pandemic, the seminars and meetings were organized online. However, it was also possible to provide training to students from Belarus through the Erasmus+ program. The data acquired was analyzed and discussed during the seminars. The Belarusian partner was responsible for the organization of the seminars at the Vetka Museum, where all participants learned about the history of the ICONS project and conservative needs.

## Figures and Tables

**Table 1:** Areas analyzed by XRF spectroscopy, localization, color, identified elements, and corresponding estimated pigments. Majority elements are shown in bold.

Area	Localization	Area	Elements	Pigments
P1	Mantle Child	Red	**Hg**, Pb, Ca, Fe, Mn, Sr	Cinnabar, gypsum
P2	Mantle Madonna	Dark red	**Fe**, Ca, Sr, Mn, Pb, Hg, Cu	Umber, cinnabar, copper-based pigment, gypsum
P3	Sleeve Child	White	**Pb**, Fe, Ca, Sr	White lead, gypsum
P4	Belt Child	Blue	**Cu**, Pb, Fe, Mn, Hg, Ca	Umber, copper-based pigment
P5	Face Child	Incarnate	**Pb**, **Hg**, Fe, Ca, Sr	White lead, cinnabar
P6	Background	Gold	**Au**, **Fe**, Si, K, Ca, Sr	Gold, clays (bolo)
P7	Halo	Gold	**Ca**, Fe, Mn, Au, Sr, Pb	Clays (bolo), gold, gypsum, white lead
P8	Preparation Dx	White	**Ca**, S, Fe, Mn, Sr, Cr, Pb	Gypsum, white lead, clays
P9	Preparation Sn	Bianca	**Ca**, S, Fe, Sr, Cr, Pb	Gypsum, white lead, clays
P10	Background Gold	Gold	**Ca**, Fe, Mn, Au, Sr, Pb	Bolus, gold, gypsum, white lead
P11	Sleeve Madonna	Blue	**Cu**, Pb, Fe, Mn, Ca, Sr	Copper-based pigment, gypsum, umber
P12	Hair Child	Brown	**Fe**, **Mn**, Ca, Sr, Hg, Pb	Umber, cinnabar
P13	Gold	Gold	**Ca**, Fe, Au, Sr, Pb	Bolus, gold, gypsum, white lead

## Data Availability

The datasets generated during and/or analyzed during the current study are available from the corresponding author on reasonable request.

## Supplementary Materials

The following are available online. Figure S1: Vis diffuse—Detail, veil and halo of Madonna. Figure S2: Vis diffuse—lower left corner, repainting. Figure S3: Diffuse vis—Detail, lipstick imprint on the right side. Figure S4: Vis grazing—Detail of the inscription showing some engraved letters. Figure S5: UV—Inscription on the left of the Madonna where an orange fluorescence is observed in correspondence with the symbol on the central letters. Figure S6: IR—Detail, face of the Madonna and Child Jesus. Figure S7: IR—Detail, Madonna and Child Jesus. Figure S8: IR: Detail, shoulder of Madonna. Figure S9: Radiography. Figure S10: Maps of the area analyzed by XRF (left) and IR (right) spectroscopy. Figure S11: IR spectra: blue P1, red P2 and pink P3.
