# Supplementary material for: Sicilian Byzantine Icons through the Use of Non-Invasive Imaging Techniques and Optical Spectroscopy: The Case of the Madonna dell’Elemosina
Source: Molecules. 2021 Dec 15;26(24):7595. doi: 10.3390/molecules26247595 (PMC8709419; doi:10.3390/molecules26247595)
Supplement: Supplementary file 1 [file molecules-26-07595-s001.zip › molecules-1498421-supplementary.pdf]

Supplementary Materials

Sicilian Byzantine Icons through the use of non-invasive imaging techniques and optical spectroscopy. The case of the *Madonna dell'Elemosina*

F. Armetta[1], G. Chirco[1], F. Lo Celso[2], V. Ciaramitaro[1], E. Caponetti[1,3], M. Midiri[4], G. Lo Re[4], V. Gaishun[5], D. Kovalenko[5], A. Semchenko[5], D. Hreniak[6] , M L Saladino[1*]

[1] *Department of Biological, Chemical and Pharmaceutical Sciences and Technologies - STEBICEF, University of Palermo, Viale delle Scienze Ed.17, Palermo (Italy)*
[2] *Department of Physics and Chemistry "E. Segre", University of Palermo, Viale delle Scienze Ed. 18, Palermo (Italy)*
[3] *Labor Artis C.R. Diagnostica S.R.L., Palermo (Italy)*
[4] *Biomedicine, Neuroscience and Advanced Diagnostics, University of Palermo, Via del Vespro 129, I-90127 Palermo (Italy)*
[5] *Francisk Skorina Gomel State University, Soviet str. 104, Gomel (Republic of Belarus)*
[6] *Institute of Low Temperature and Structure Research, Polish Academy of Sciences (ILT&RS PAS), ul. Okólna 2, Wrocław (Poland)*

**corresponding author: marialuisa.saladino@unipa.it*

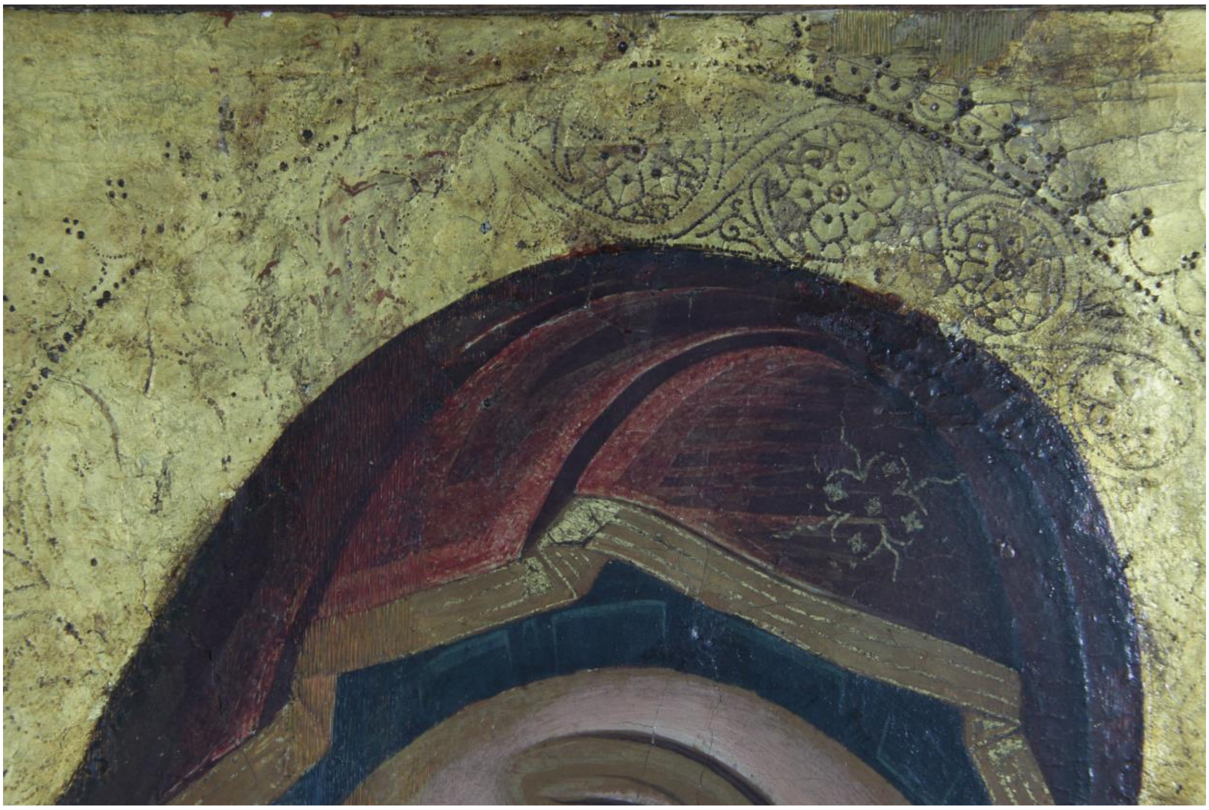

Figure S1 - Vis diffuse - Detail, veil and halo of Madonna.

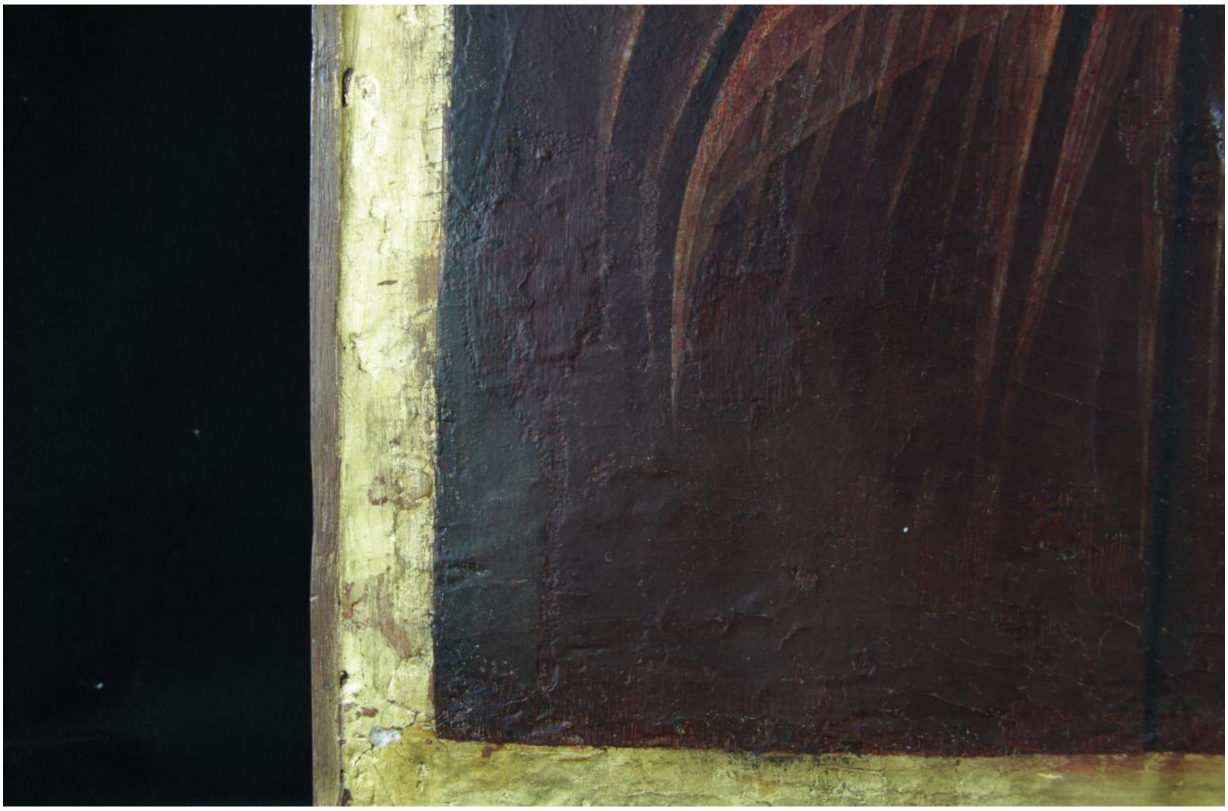
Figure S2- Vis diffuse - lower left corner, repainting.

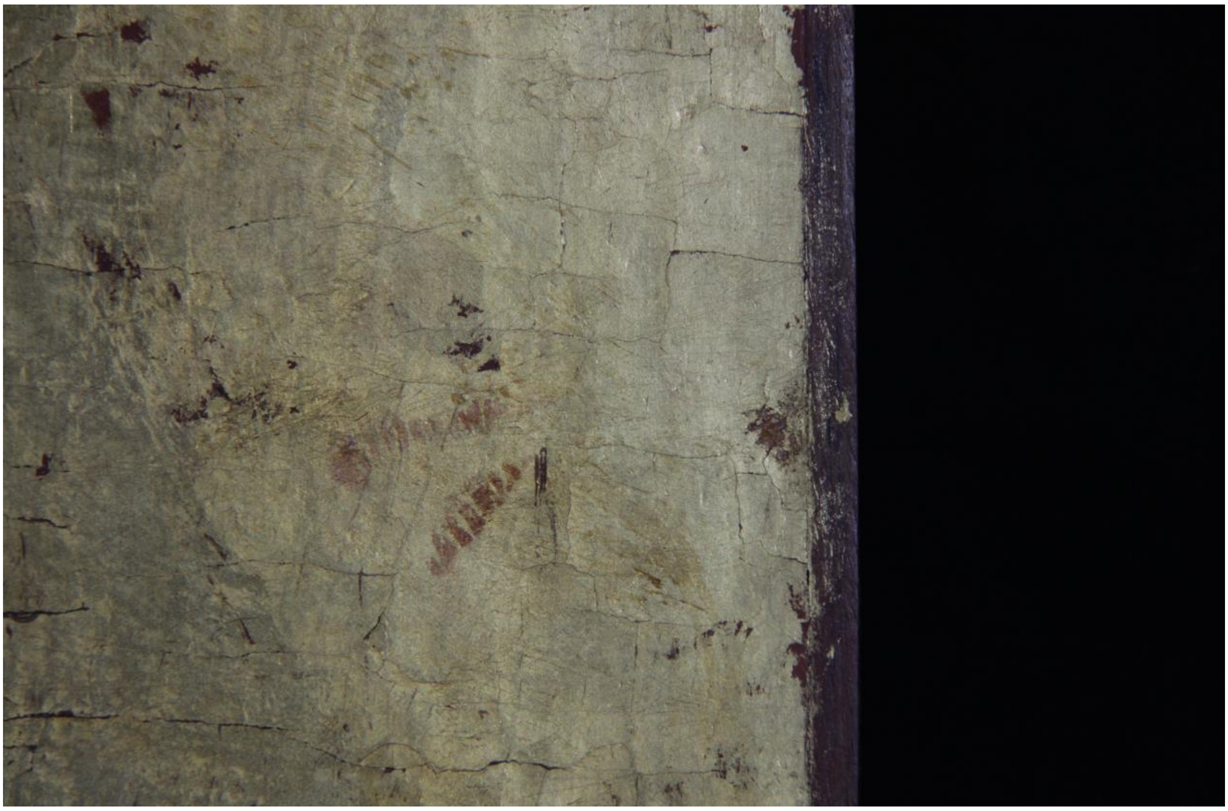
Figure S3- Diffuse vis - Detail, lipstick imprint on the right side.

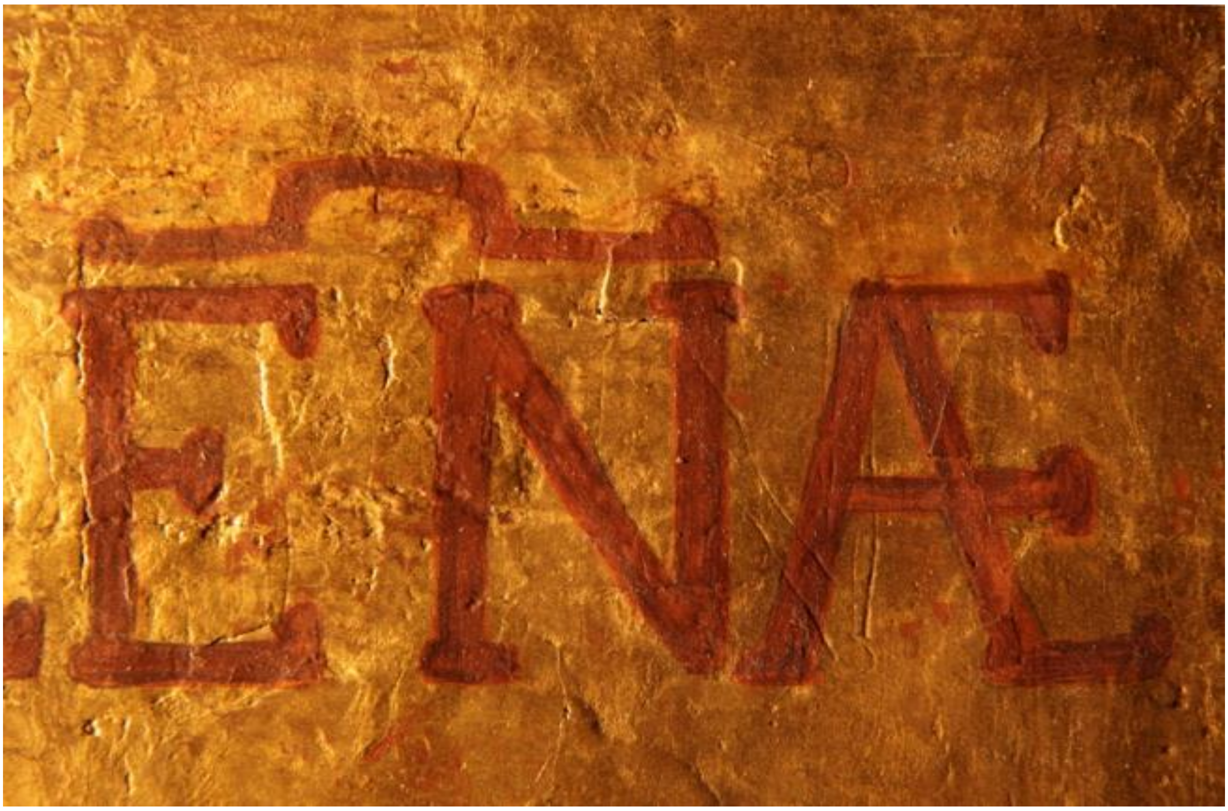

Figure S4 - Vis grazing - Detail of the inscription showing some engraved letters.

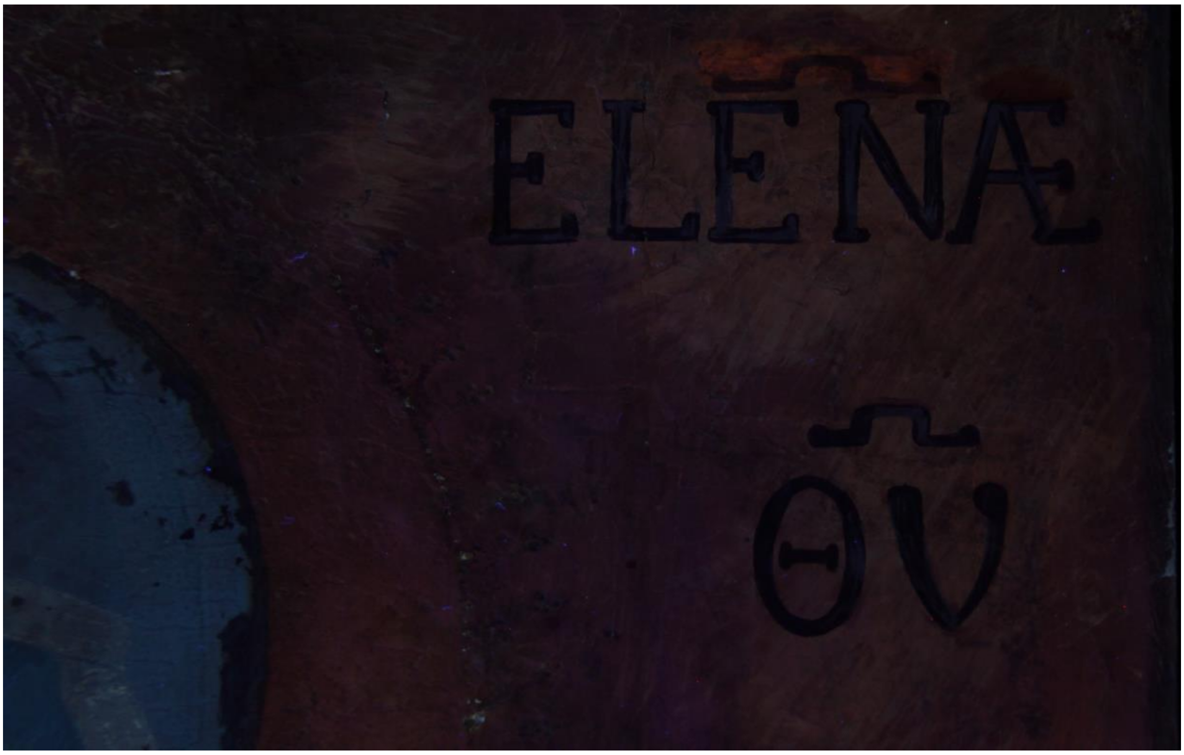

Figure S5 - UV - Inscription on the left of the Madonna where an orange fluorescence is observed in correspondence with the symbol on the central letters.

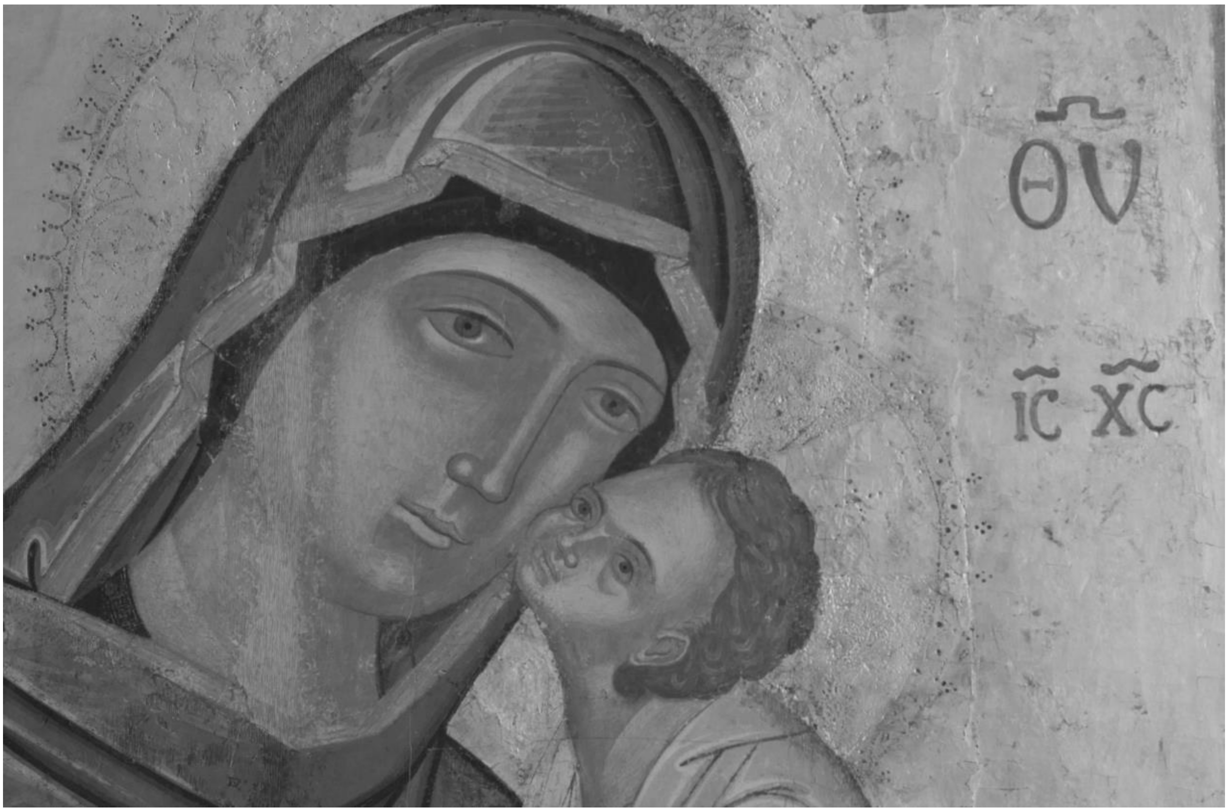

Figure S6 - IR - Detail, face of the Madonna and Child Jesus.

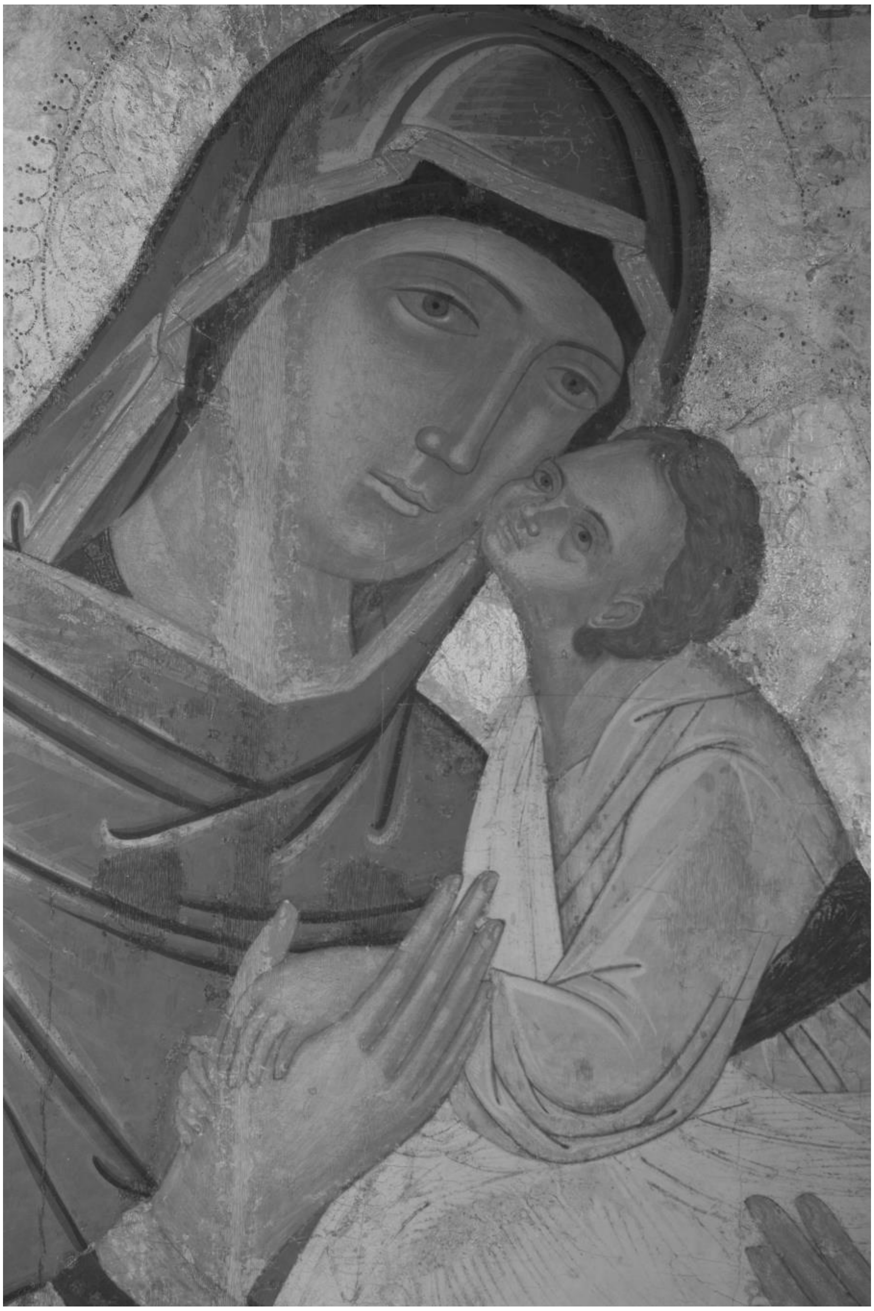

Figure S7 - IR - Detail, Madonna and Child Jesus.

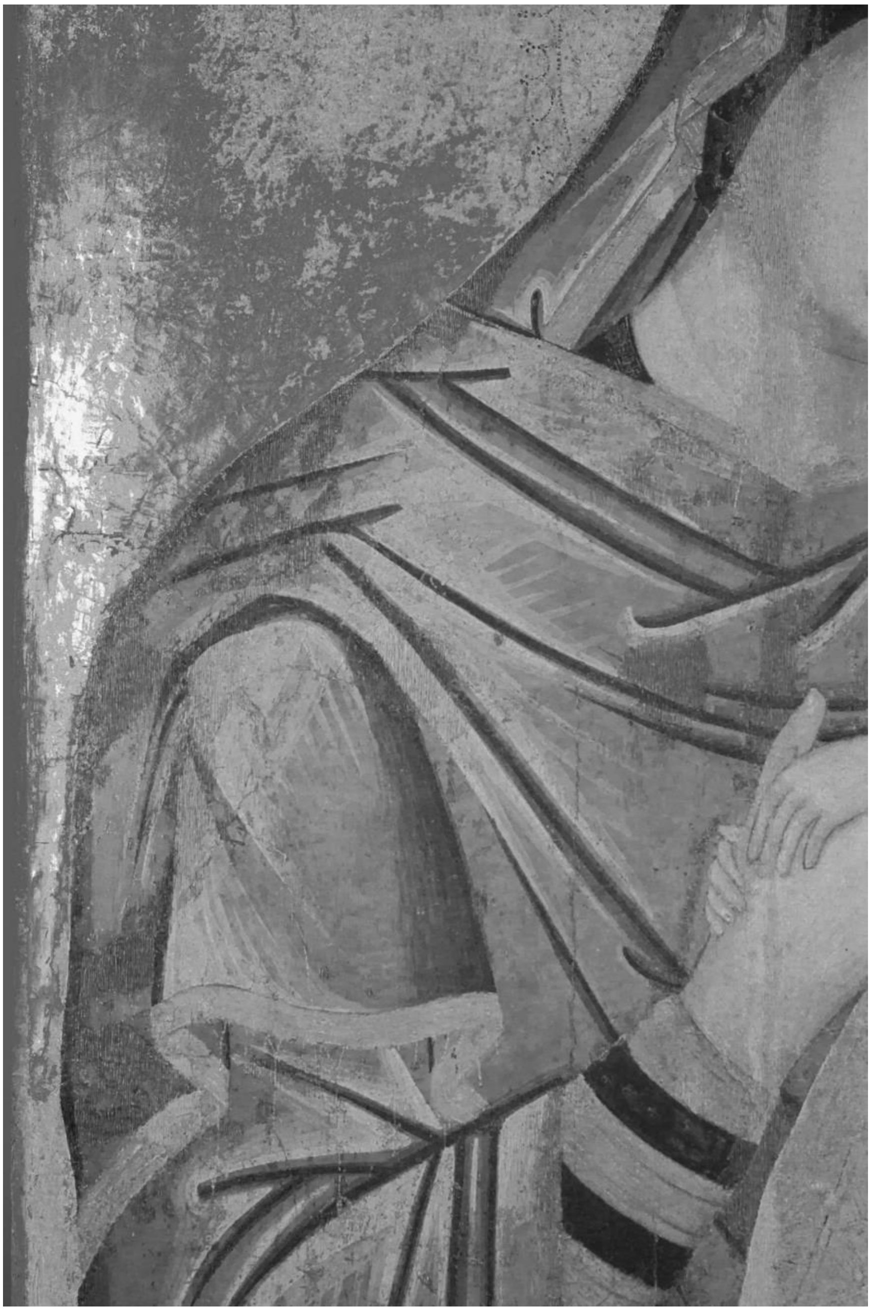
Figure S8 - IR - Detail, shoulder of Madonna.

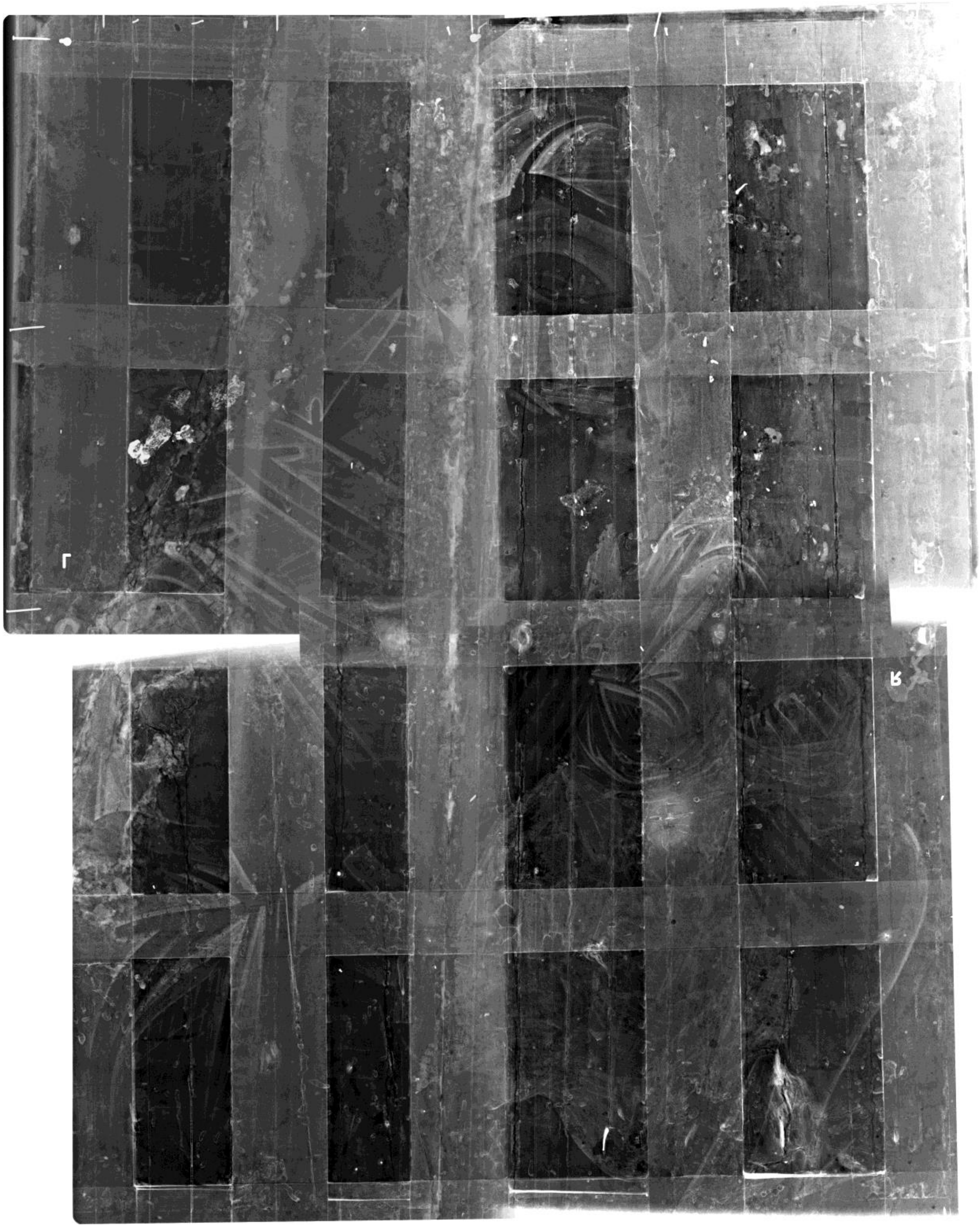

Figure S9. Radiography.

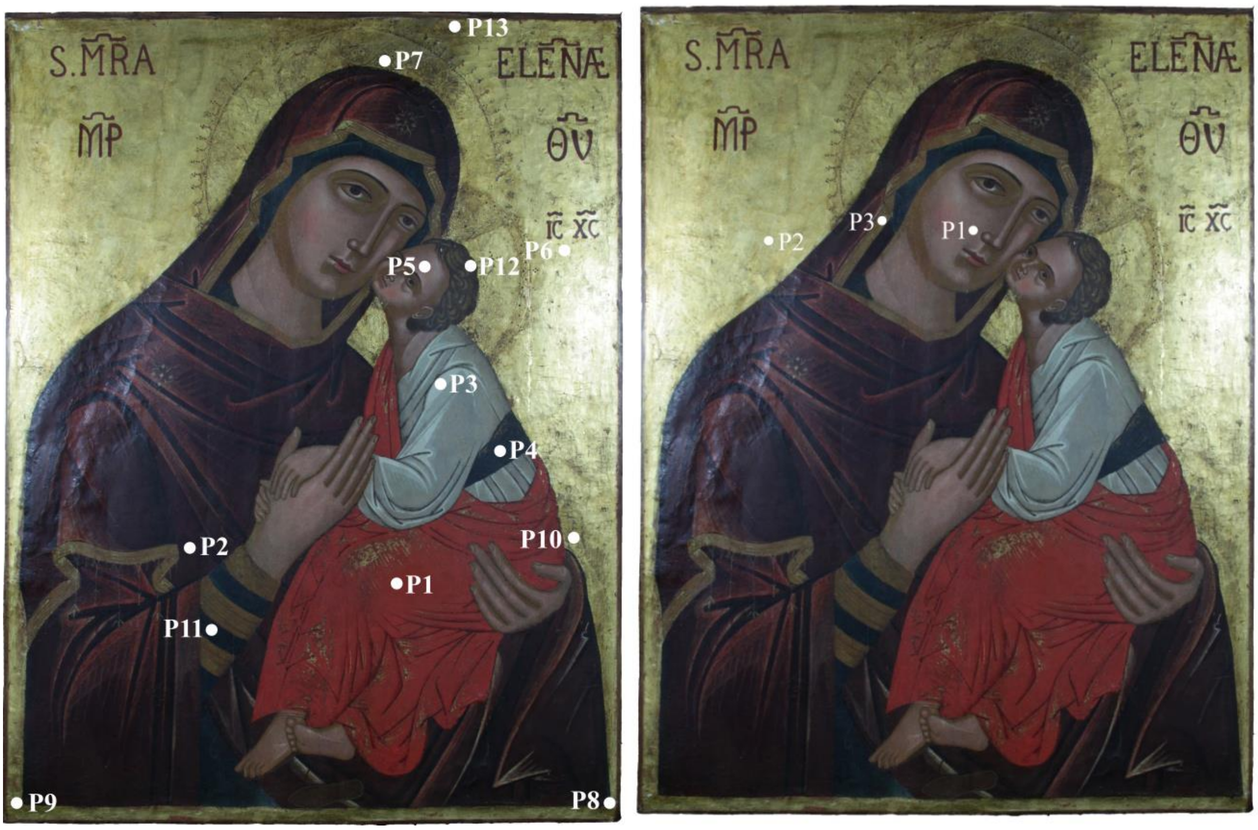

Figure S10. Maps of the area analyzed by XRF (left) and IR (right) spectroscopy.

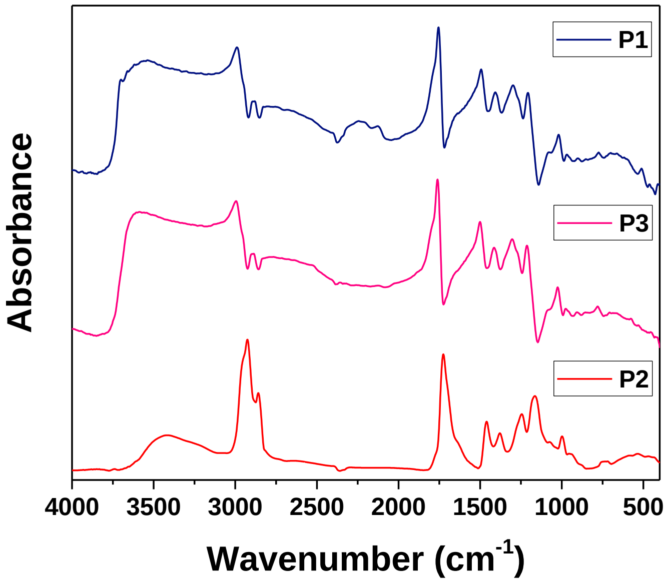

Figure S11. IR spectra: blue P1, red P2 and pink P3.
